# Influence of Muscle Mass and Strength on Bone Mineralisation with Consideration of Sclerostin Concentration

**DOI:** 10.3390/biomedicines11061574

**Published:** 2023-05-29

**Authors:** Martyna Patalong-Wójcik, Anna Golara, Katarzyna Zając, Alicja Sokołowska, Mateusz Kozłowski, Aleksandra Tołoczko-Grabarek, Mariola Krzyścin, Agnieszka Brodowska, Agnieszka Janiec, Aleksandra Myszka, Aneta Cymbaluk-Płoska, Elżbieta Sowińska-Przepiera

**Affiliations:** 1 Department of Endocrinology, Metabolic and Internal Diseases, Pomeranian Medical University in Szczecin, UniiLubelskiej 1, 71-252 Szczecin, Poland; patalongmartyna@gmail.com (M.P.-W.); krzyscin@tlen.pl (M.K.); taga3@wp.pl (A.J.); myszka-ola@hotmail.pl (A.M.); elzbieta.sowinska.przepiera@pum.edu.pl (E.S.-P.); 2 Department of Reconstructive Surgery and Gynecological Oncology, Pomeranian Medical University in Szczecin, Al. Powstańców Wielkopolskich 72, 70-111 Szczecin, Poland; kasiazajac24@onet.pl (K.Z.); alasok99@gmail.com (A.S.); mtkoozo@gmail.com (M.K.); aneta.cymabluk@gmail.com (A.C.-P.); 3 Department of Genetics and Pathology, Pomeranian Medical University in Szczecin, 71-252 Szczecin, Poland; otjg@interia.pl; 4 Department of Gynecology, Endocrinology and Gynecological Oncology, Pomeranian Medical University in Szczecin, UniiLubelskiej 1, 71-252 Szczecin, Poland; agabrod@wp.pl; 5 Pediatric, Adolescent Gynecology Clinic, Department of Gynecology, Endocrinology and Gynecological Oncology, Pomeranian Medical University in Szczecin, UniiLubelskiej 1, 71-252 Szczecin, Poland

**Keywords:** bone mineralisation, sclerostin, muscle mass, osteocyte, muscle strength

## Abstract

Osteoporosis is a disease characterised by a reduction in bone strength due to increased porosity and impaired mineralisation. In our study, we investigated whether muscle strength and mass exert a significant effect on bone mineral density in young adult women. We also tested whether sclerostin can be used as an indicator in the assessment of bone mineralisation. The study included 111 patients. All patients had their bone mineral density determined in the L1–L4 section of the lumbar spine and in the whole skeleton. The parameters of fat mass (FM), lean body mass (LBM) and visceral fat mass (VF) were also determined. Metabolic activity of osteocytes was assessed by measuring the serum sclerostin concentration. There was a statistically significant association of both hands’ muscle strength with all parameters expressing bone mineralisation. A statistically significant relationship was also obtained between BMD L1–L4 and the body mass components (FM, LBM). Sclerostin levels in the study did not differ between groups with normal and reduced bone mineral density. Muscle strength assessment may be a potential exponent of reduced bone mineral density, also used clinically in young adult women. The utility of sclerostin in the clinical assessment of bone mineralisation has not been demonstrated.

## 1. Introduction

Peak muscle mass is attained at approximately 25 years of age, but unlike bone mineralisation, there is little decline until the 5th decade of life. Thereafter, there is a gradual decrease in muscle mass, with a loss of approx. 30% by the age of 80 [1,2]. Body mass has two main components: fat body mass (FBM) and lean body mass (LBM), about half of which corresponds to muscle mass [3,4]. One of the components of LBM is bone mineral content (BMC), which accounts for approximately 5% of its value. The exception is premenopausal women, in whom this value is approximately 17–29% higher. Mechanical stimulation of the skeletal system by muscles consists of two components. The first is the muscle mass that constitutes the load on the skeleton, while the second depends on the quality of the muscle tissue, i.e., the force generated by the muscles [5]. Evidence of the impact of muscle mass was obtained, among other sources, from studies of post-stroke patients, where low BMC, BMD and fat-free tissue content were demonstrated in limbs with paresis [6]. There was a strong positive correlation between bone mass and muscle strength [7]. Muscle strength is also thought to be a BMD determinant independent of muscle size [8]. In a study involving a large, ethnically diverse and age-diverse group of Asian women, it was shown that patients with sarcopenia were twice as likely to have osteoporosis/osteopenia and muscle strength (measured by handgrip strength) correlated positively with BMD in every body region [9]. The direct effect of mechanical loading on bone formation, according to mechanostat theory, explains the relationship between body mass and bone mineral density.

Positive correlation also exists between body fat mass and BMD, which is related, in addition to mechanical loading, to the effect of body fat on oestrogen metabolism [10,11,12]. The positive effects of adipose tissue on the skeleton are partly abolished by its negative effects. This is because greater adipose tissue mass is associated with an increase in the pro-inflammatory proresorptive cytokines IL-6 and TNF-alpha and increased bone marrow fat conversion [13]. The adipokines, including adiponectin and leptin, have been demonstrated to regulate inflammatory immune responses in cartilage. Obese people and animals show higher levels of serum tumour necrosis factor-alpha (TNF-α), interleukin-1 beta (IL)-1β and IL-6, all of which are produced by macrophages derived from adipose tissue [14].

The effect of muscles on bone is not only related to mechanical interaction, but there is also a biochemical connection between these tissues. Muscles produce trophic factors called myokines, which can affect bone through auto-, para- and endocrine pathways [15]. The first molecule identified as a myokine was myostatin, a protein belonging to the TGF-beta family. It is secreted primarily by muscle cells [16] and is a negative regulator of muscle mass (causes increased protein degradation, decreased protein production and inhibition of myogenesis) [17].

In addition to significant hypertrophy and muscle strength, mice lacking the myostatin gene also showed increased bone density and mineral content and osteogenesis in response to exercise [18,19]. In contrast, an in vitro study showed inhibition of osteoprogenitor cell differentiation and proliferation, which was MSTN-concentration-dependent [20]. Other studies have also observed suppression of bone mineralisation [21]. Sclerostin is a glycoprotein that inhibits bone formation processes. It is the most important factor reducing osteoblast function [22]. Its concentrations increase in direct proportion to age: it has been shown that sclerostin concentrations are significantly lower in women aged 30–34 years compared to the group aged 35–40 years [23,24,25,26]. Bone damage and mechanical factors (e.g., exercise) inhibit sclerostin secretion, leading to a predominance of anabolic processes [27]. In contrast, the action of overly low forces generates an increase in its expression and secretion, as demonstrated in studies carried out on patients with spinal cord injuries [23,28,29].

Sclerostin also increases the expression of proteolytic enzymes in osteoclasts [30]. The original study showed a direct correlation between BMD and sclerostin concentration, in groups of healthy women and in people with spinal cord injury, and posited that sclerostin concentration could complement or even replace densitometric testing [23,24]. The relationship between BMD and sclerostin is most likely related to a higher number of sclerostin-producing osteocytes, reflecting bone mass [31]. The aim of the study was to assess the correlation of muscle mass and strength with bone mineralisation. We also investigated the correlation of sclerostin concentrations with bone mass, muscle strength and mineralisation and assessed whether sclerostin could be useful in clinical practice as an exponent of bone mineralisation.

## 2. Results

### 2.1. Characteristics of the Group

The characteristics of all the data collected for the calculations are shown in Table 1.

### 2.2. Bone Mineralisation

The characteristics of the patient groups divided according to the L1–L4 BMD Z-score values (group A, Z-score > −1.0, and group B, Z-score < −1.0), and their comparison is shown in Table 2.

There were no statistically significant differences in muscle strength between the groups with normal (A) and reduced (B) bone mineral density.

Spearman’s rank correlation coefficients (R) between BMD L1–L4 bone mineral density and the studied parameters are shown in Table 3.

Significant positive correlations were observed between BMD L1–L4 bone mineral density and right hand muscle strength (weak positive correlation, R = 0.3090; *p* = 0.0009), left hand muscle strength (weak positive correlation, R = 0.3683; *p* = 0.0000), fat mass (weak positive correlation, R = 0.2788; *p* = 0.0030), lean body mass (very strong positive correlation, R = 0.9214; *p* = 0.0000) and fat–lean ratio (weak positive correlation, R = 0.2042; *p* = 0.0315).

The data analysis showed no differences of statistical significance in the bone mineralisation parameters (BMD total, BMD L1–L4, Z-score L1–L4, BMC) between the specified sclerostin concentration groups (I, II, III, IV). The results also remained statistically insignificant when calculated using sclerostin concentration as a quantitative variable.

### 2.3. Muscle Mass and Strength

**Figure 1:**
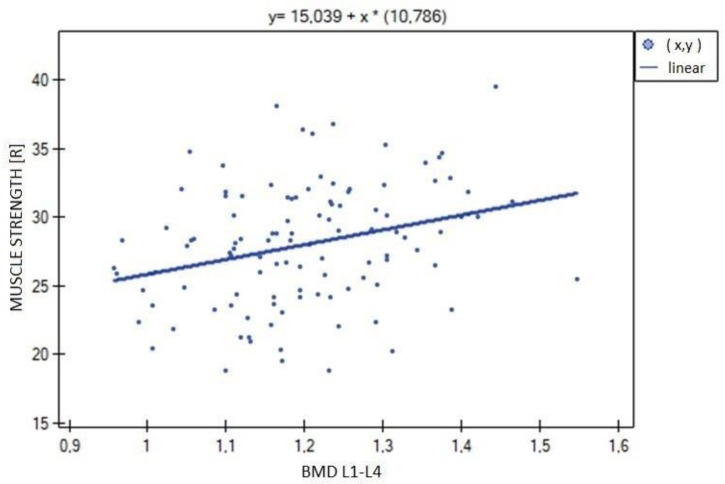
Relationship between bone mineral density (BMD) L1–L4 and muscle strength of the right hand (R = 0.3090, *p* = 0.0009).

**Figure 2:**
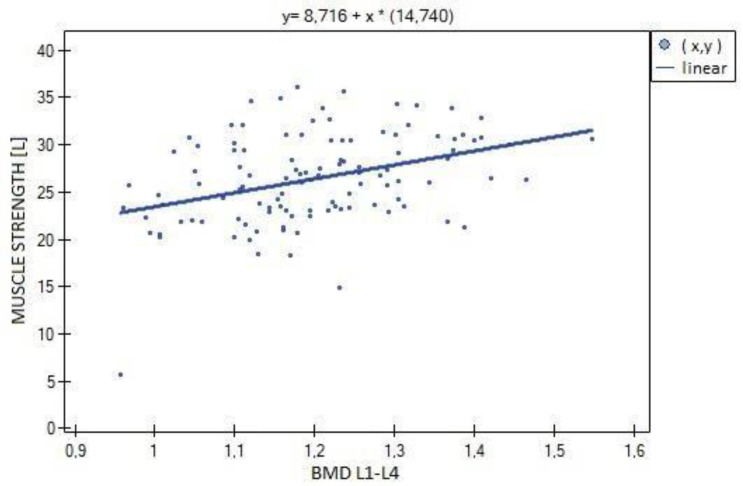
Relationship between bone mineral density (BMD) L1–L4 and muscle strength of the left hand (R = 0.3683, *p* = 0.0000).

**Figure 3:**
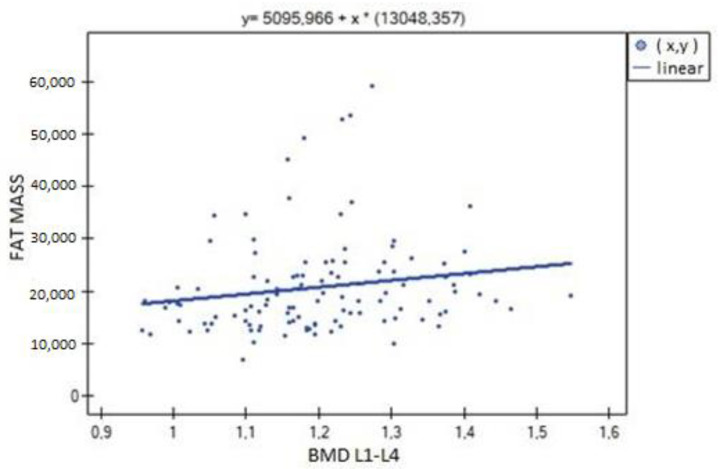
Relationship between bone mineral density (BMD) L1–L4 and fat mass (R = 0.2788, *p* = 0.0030).

**Figure 4:**
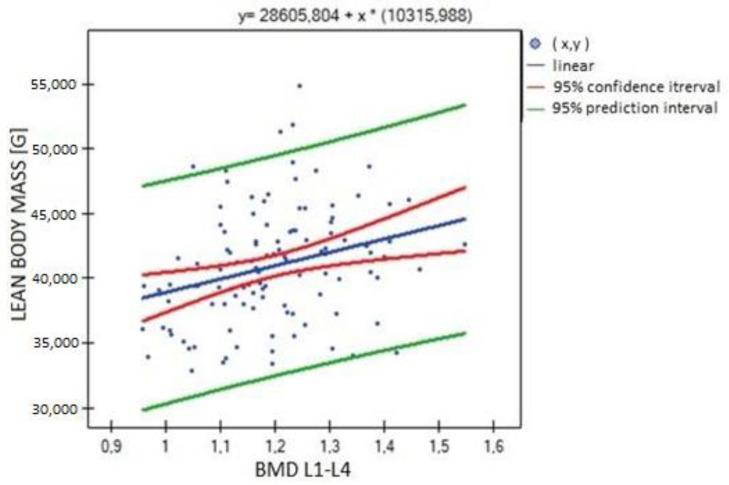
Relationship between BMD L1–L4 bone mineral density and lean body mass (R = 0.9214, *p* = 0.0000).

**Figure 5:**
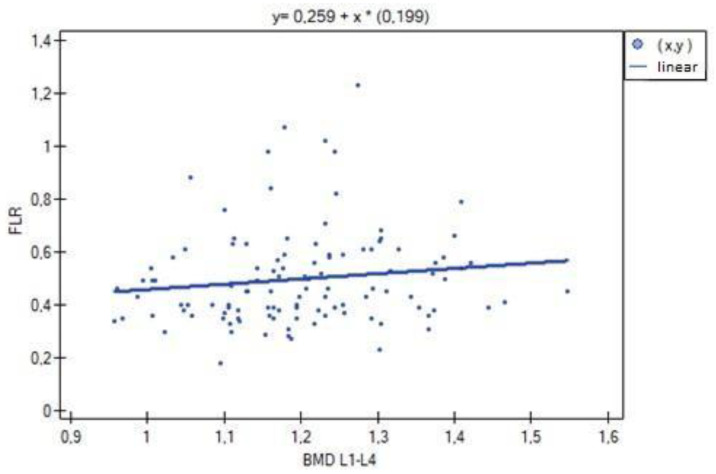
Relationship between bone mineral density (BMD) L1–L4 and FLR (R = 0.2042, *p* = 0.0315).

In the analysis performed between BMD values L1–L4 and muscle strength of both upper limbs, a weak, statistically significant positive correlation was found (right side R = 0.3090, *p* = 0.0009; left side R = 0.3683, *p* = 0.0000). The correlations are shown in Figure 1 and Figure 2. There were no statistically significant differences in muscle strength between groups with normal (A) and reduced (B) bone mineral density. There was also no correlation between bone density and the body mass components studied. There was no statistical significance for fat-free mass (*p* = 0.7054) or fat mass (*p* = 0.4325). Statistically significant relationships were obtained between BMD L1–L4 and the body mass components fat mass (weak positive correlation, R = 0.2788, *p* = 0.0030) and lean body mass (very strong positive correlation, R = 0.9214, *p* = 0.0000) and their FLR ratio (weak positive correlation, R = 0.2042, *p* = 0.0315). A similar relationship was not found for visceral fat mass VF (R = 0.1586, *p* = 0.0963). The correlations between BMD L1–L4 and body mass components are shown in Figure 3, Figure 4 and Figure 5.

The results obtained for the correlation of right- and left-hand muscle strength are shown in Table 4.

Significant positive correlations are observed between lean body mass (LBM) and total bone mineral density (R = 0.615; *p* = 0.000), bone mineral density of the L1–L4 segment (R = 0.301; *p* = 0.001), bone mineral content (R = 0.771; *p* = 0.000), fat mass (R = 0.526; *p* = 0.000), fat–lean ratio (R = 0.286; *p* = 0.002) and visceral fat mass (R = 0.303; *p* = 0.001).

Significant positive correlations also existed between left hand muscle strength and total bone mineral density (weak positive correlation, R = 0.3246; *p* = 0.0005), bone mineral density of the L1–L4 segment (weak positive correlation, R = 0.3683; *p* = 0.0001), bone mineral density Z-score (weak positive correlation, R = 0.3086; *p* = 0.0010), bone mineral content (weak positive correlation, R = 0.3864; *p* = 0.0000) and lean body mass (weak positive correlation, R = 0.3618; *p* = 0.0001). Only for the parameter BMD total was no statistical significance obtained (*p* = 0.0759).

There were no statistically significant differences in muscle strength of the right hand (*p* = 0.1134) and left hand (*p* = 0.3735) between patients in the four sclerostin concentration groups.

### 2.4. Body Composition

The results obtained for the correlation of lean body mass are shown in Table 5.

**Figure 6:**
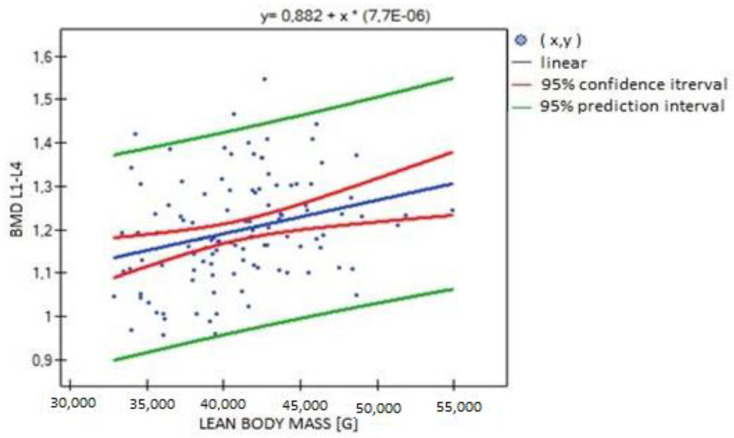
Relationship between lean body mass (LBM) and BMD L1–L4 (R = 0.301, *p* = 0.001).

**Figure 7:**
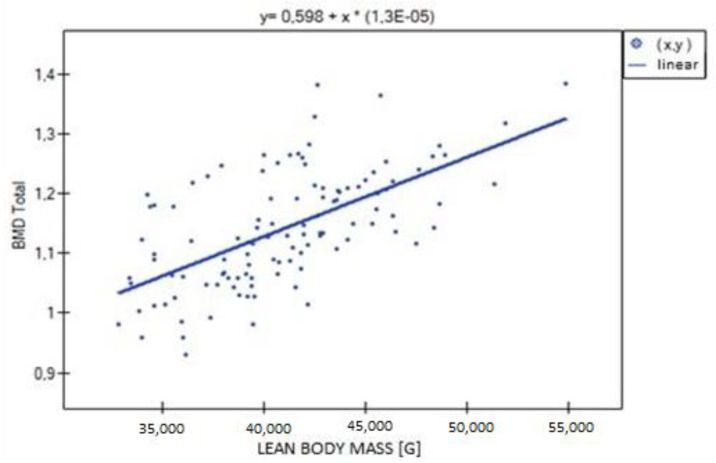
Relationship between lean body mass (LBM) and total BMD (R = 0.615, *p* = 0.000).

**Figure 8:**
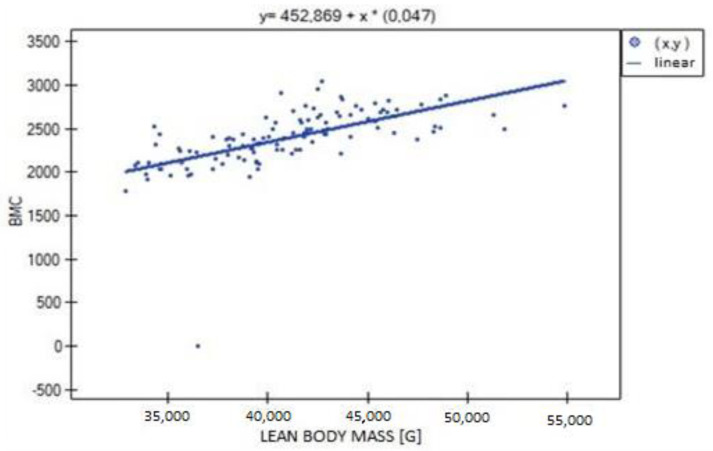
Relationship between lean body mass (LBM) and BMC (R = 0.771, *p* = 0.000).

Significant positive correlations were observed between lean body mass (LBM) and all parameters expressing bone mineralization (total bone mineral density (strong correlation, R = 0.615; *p* = 0.000), bone mineral density of the L1–L4 segment (weak correlation, R = 0.301; *p* = 0.001), bone mineral content (strong correlation, R = 0.771; *p* = 0.000)), except for Z-score L1–L4, for which no statistical significance was obtained (*p* = 0.814). There was significant positive correlation between LBM and fat mass (moderate correlation, R = 0.526; *p* = 0.000), fat–lean ratio (weak correlation, R = 0.286; *p* = 0.002) and visceral fat mass (weak correlation, R = 0.303; *p* = 0.001) (Figure 6, Figure 7 and Figure 8).

Statistically significant weak positive correlations were found between muscle strength of both hands and muscle tissue mass: left side (R = 0.3618, *p* = 0.0001), right side (R = 0.2434, *p* = 0.0110). No such relationship was found for fat mass.

In the comparative analysis of sclerostin concentration and lean body mass, there was no statistical significance (*p* = 0.1328) by group. In the analysis of sclerostin as a numerical variable, the relationship remained at the limit of statistical significance (*p* = 0.0509). The value of fat mass (FM) also did not differ significantly between groups, but a statistically significant relationship was found between sclerostin concentration and visceral fat mass (VF, *p* = 0.0345). The relationship was only present when patients were divided into groups.

### 2.5. Sclerostin

**Figure 9:**
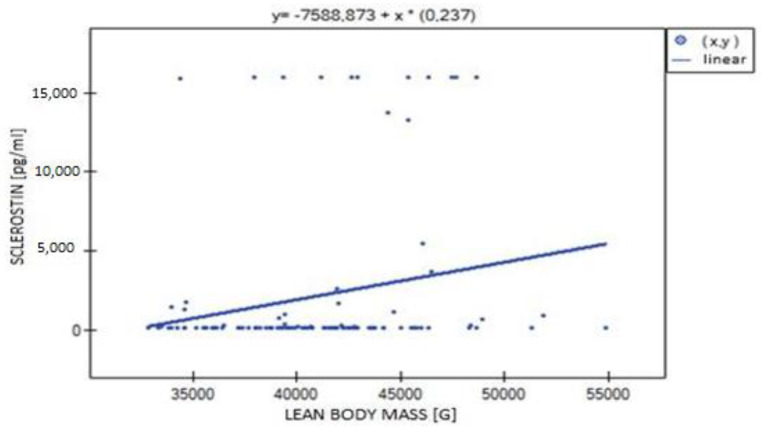
Relationship between lean body mass (LBM) and sclerostin (R = 0.186, *p* = 0.051).

The data analysis showed no difference of statistical significance in sclerostin concentration as a quantitative variable between the groups with normal and reduced bone mineral density. There was also no correlation between sclerostin concentration and BMD L1–L4 (Figure 9). There were no differences in bone mineral density indices between patient groups determined by sclerostin concentration (I, II, III, IV).

The correlation analysis between sclerostin concentration and hand muscle strength showed no statistical significance for either limb. There was a very weak positive correlation between LBM and sclerostin concentration, but the result remained at the limit of statistical significance (*p* = 0.051) (Figure 9).

## 3. Discussion

The aim of the study was to determine whether muscle strength and mass have a significant impact on bone mineral density in young adult women and whether sclerostin concentration is useful in its assessment. On the base of that knowledge, we aimed to identify clinical and biochemical markers that are risk factors for low bone mineralization in young adult women.

In our study, we showed an unequivocal positive relationship between muscle strength in both limbs and parameters assessing bone mineralisation: BMD total, BMD L1–L4, Z-score L1–L4 and BMC. The obtained results are consistent with data available in the literature.

A positive association between muscle strength (including grip strength) and bone density in young adults was also demonstrated in a study by US researchers, in which correlations varied according to the body region studied, but the study’s final conclusion confirmed that muscle strength is an independent predictor of BMD values [32].

In the United States, a large data analysis (National Health and Nutrition Examination Survey—NHANES) was conducted in which muscle strength values measured by handgrip strength and BMD values of the lumbar spine (L1–L4) and proximal end of the femur obtained from participants of both sexes aged 40–80 years were assessed. The results support the conclusions of this study: there was an unequivocal correlation of handgrip strength with L1–L4 and femoral neck BMD in the premenopausal female group. The results also remained significant when patients were subdivided according to body weight, which made it possible to rule out cross-over effects of body weight and muscle on the relationships studied [33]. When comparing the results of our study, it should be borne in mind that the data obtained in the American analysis included patients more than 10 years older than the women enrolled in this study. Although they were still menstruating, their hormonal status may have been different (perimenopause), which may have significantly modified the observed relationships.

Slightly different results were obtained in a Swedish cross-sectional study in which statistically significant relationships were found between whole-body bone mineral density (BMD total) and muscle strength in a group of women, but only in the lower limb. No such relationship was shown for hand grip strength, nor was it observed in the male study participants [34].

In 2020, however, a meta-analysis was published in which an attempt was made to standardise knowledge on the relationship between muscle strength and mineralisation in a group of patients before/during the acquisition of peak bone mass. Studies on children, adolescents and young adults were included in the meta-analysis. Studies measuring muscle strength of both upper limbs and lower limbs were evaluated. The results unequivocally confirmed that muscle function positively influences BMD and BMC both locally and overall systemically. The relationship between BMC and upper limb strength was significant, with all correlations showing greater strength in the boys’ group [35]. In this discussion, it is necessary to cite the study published by Pratt J. et al. in which the highest muscle strength, irrespective of gender, was obtained in the 30–39 age group. In women, a significant decrease appeared around 45 years of age, and it appeared in men around 5 years later. Interestingly, no parallel decrease in skeletal muscle index (SMI) was observed, which allows us to conclude that the loss of muscle quality starts with muscle strength. In addition, the authors attempted to set borderline low values for handgrip strength, which were set at <33.95 kg for men and <21.68 kg for women [36]. We show in this study an unequivocal positive association between muscle strength in both limbs and these parameters.

The results obtained in this study and the literature data cited above clearly confirm how important good-quality muscle tissue is for bone health.

Researchers have repeatedly asked the question of which component of body weight has a greater impact on bone wellbeing. The results obtained in numerous studies have been inconsistent and contradictory. It would seem that if the influence was solely an effect of weight load, the effect of FM and LBM should be equal. An attempt to systematise knowledge in this area was made by a group of researchers (Ho-Pham et al.) in a meta-analysis, which showed a positive association of both body mass components with bone mineral density but unequivocally proved that there was a greater effect of lean soft tissue. Moreover, this regularity was independent of age, gender or ethnicity. In the group of postmenopausal women, an equivalent effect of both components was found, which allows us to assume that in the premenopausal period, it is the quality of muscle, or indirectly physical activity, that will be more important for gaining and maintaining bone mass [37]. The increased impact of FM after menopause is most likely due to a protective effect related to the oestrogen metabolism taking place within it.

In the analysis presented in the present study, there was a positive correlation between lean body mass and the studied parameters expressing bone mineralisation (total BMD, BMD L1–L4, BMC), except for the Z-score L1–L4, for which no statistical significance was obtained. In addition, LBM positively correlated with muscle strength tested, which, as described above, is an independent factor for a positive prognosis of the skeleton.

The results obtained in this study can be compared to the analysis carried out by the Brazilian researchers who conducted an observational study: they assessed bone density and body mass components in female patients aged 18 and then 22 years. In women, the value of lean body mass was shown to have the greatest effect on bone density gain. This effect included both whole-body and femoral neck BMD. It was also confirmed that despite the positive correlation between BMD and BMI and both body mass component indices (FMI and LMI), it was fat-free mass that showed the greatest effect on the development of peak bone mass [38]. Admittedly, our study did not show a correlation with fat mass, but within the influence of fat-free mass, the results and conclusions are consistent. Another large-cohort study is the observation by Denova-Gutiérrez E. et al., where the effect of muscle tissue on mineralisation was shown to be increased at Tanner stage 3 of puberty [39]. The researchers justify the detection of such a point in girls with an increase in sex hormones, which have a permissive effect on the bone’s response to loading. Another publication worth citing is another part of the NHANES study, whose results showed a sex-, age- and race-independent positive effect of LBM on BMD. Fat mass, on the other hand, had a negative effect on bone density, but the results varied depending on the method of analysis adopted [40]. The studies collected above exploring the relationship between muscle mass and bone mineralisation remain consistent with the results obtained in this paper.

Sclerostin is a protein secreted by osteocytes that is an inhibitor of the canonical Wnt-beta catenin pathway, thereby inhibiting bone formation [41]. The anti-sclerostin antibody romosozumab was approved by the European Medicines Agency (EMA) in 2019 for the treatment of osteoporosis [42]. Sclerostin appears to be a promising marker for assessing bone mass. The availability of data on young adults is scarce. We did not demonstrate a significant association of sclerostin concentration with any of the parameters studied: bone density, mass or muscle strength. For fat-free tissue mass, the relationship remained at the limit of statistical significance. The association of sclerostin concentration with visceral fat (VF) mass was demonstrated. J. Coulson et al. analysed the association of bone mineralisation density, age, sclerostin concentration, osteoprotegerin and dickkopf-1 protein (DKK1). In the MYOAGE cross-sectional cohort study, higher concentrations of sclerostin, DKK1 and OPG were found in older adults compared to young adults. Sclerostin levels correlated positively with bone mineral density only in the elderly group; no such relationship was found in young adults. It is suspected that mature osteocytes, present in mineralised bone tissue, inherently produce higher amounts of sclerostin, accounting for such a correlation. On the other hand, perhaps an age-related decrease in glomerular filtration rate results in the persistence of increased sclerostin values [43]. This explanation appears to have a strong basis, in view of the fact that sclerostin levels have been shown to be increased in both patients with acute kidney injury [44] and patients with chronic kidney dysfunction [44,45,46]. Similar findings in relation to the positive correlation of sclerostin concentration and bone density were demonstrated by Sharma-Ghimire P. et al. In this study, young adults (20–30 years) and middle-aged premenopausal women (35–45 years) were compared to each other. This analysis also revealed a positive association of sclerostin concentration with age. Sclerostin concentration was positively associated with bone density, not only in the group of middle-aged women but in the entire study population [47].

An observation carried out at the Mayo Clinic yielded slightly different results. This study also showed a relationship between sclerostin and age. There was no correlation between sclerostin and TTBMC (total body bone mineral content) in young women (20–39 years), a moderate correlation in the middle-aged group (40–59 years) and the strongest correlation in older women (over 60 years). These results were contrary to those expected. In addition, for a given TTBMC value, younger women had lower sclerostin levels. Thus, the suspicion of increasing osteocyte activity with age was put forward, which would explain in part the impaired bone formation in the elderly. Bone mineral density expressed by TTBMD and aBMD was not related to sclerostin in the group of youngest women, but such a positive relationship appeared already in middle and old age. In contrast to Ardawi MSM et al., the association of sclerostin with bone turnover markers was not confirmed [24]. Another interesting study is that by Amrein K. et al., where a group of premenopausal women were compared to men. In the male group, sclerostin concentrations were 25 per cent higher, whereas after analysis adjusted for, among other things, skeletal size, the differences became insignificant. In addition, sclerostin concentrations were found to be positively correlated with BMI and body fat mass, which partly coincides with the relationships shown in this study. Our study showed a positive correlation of sclerostin with visceral fat (VF) mass. Similar results were obtained by Amrein et al. [48]. This association could suggest an additional endocrine function of sclerostin, other than a local action within bone tissue.

Our study also has its limitations. The first of the potential limitations of the study is the imperfect structure of the study group. Initially, women were recruited into the study as the study and control groups. However, a thorough assessment of the patients of the Outpatient Clinic and the analysis of their health revealed a lack of chronic diseases and a lack of organic causes of the reported temporary menstrual disorders found in the course of diagnostics. Moreover, when comparing the two groups, no significant differences were found in anthropometric and laboratory parameters or in the assessment of bone density, body composition and muscle strength. Thus, all participants were included in one study group.

In the context of sclerostin concentration, the study also seems to be limited by the lack of assessment of creatinine concentration and eGFR assessment in the examined women. Literature data report higher levels of sclerostin in patients with renal insufficiency. Assessment of renal function in this study would allow us to deepen our knowledge about the relationship between sclerostin concentration and kidney function in healthy people.

The data obtained from the presented study may provide a basis for changing the clinical management of young adult female patients.

They confirm the need for regular physical activity in young adult women for the proper development of muscle mass and strength, leading to proper bone stimulation and the acquisition of bone mass.

The awareness of the need to acquire and maintain the highest possible bone mass, which will be a safeguard for later years, obliges an active search for women at risk of lower bone mass. It may be clinically useful to incorporate muscle strength testing into the assessment of a patient’s overall health. Measuring grip strength using a hand dynamometer is a simple, inexpensive procedure that requires no preparation on the part of the patient. It is also not limited by almost any potential disabilities (apart from disorders within the upper limbs). As can be seen from the data cited above, the results provide important information about the condition of both the muscular system and bones.

At present, the clinical usefulness of determining blood sclerostin levels as a marker of bone mineralisation is not established.

## 4. Materials and Methods

### 4.1. Participation in the Study

The study initially enrolled 130 women who were patients of the Gynaecological Endocrinology Outpatient Clinic of the Department of Endocrinology, Metabolic Diseases and Internal Medicine at the Pomeranian Medical University in Szczecin, who sought counselling because of transient menstrual disorders, of which organic causes of the reported abnormalities were excluded. The programme also included 103 female volunteers with no reported menstrual cycle problems.

Inclusion criteria were age 20–30 years, Caucasian females, history of normal puberty, no chronic treatment, no significant abnormalities in physical examination, and written patient consent. Exclusion criteria were endocrine diseases affecting bone mineralisation (e.g., thyroid diseases, premature ovarian function suppression—POF), severe systemic diseases affecting bone mineralisation (rheumatological diseases—RA, severe lung diseases, gastrointestinal disorders—inflammatory bowel diseases, diagnosed coeliac disease, diabetes mellitus, kidney diseases, etc.), genetic disorders or metabolic defects associated with bone mineralisation disorders, mobility disability confirmed by history, low birth weight or prematurity, at least one episode of eating disorders, impaired growth and weight gain, participation in competitive sports affecting bone mineralisation, long-term use of stimulants and drugs affecting bone metabolism and incomplete follow-up period. Based on the above criteria, 111 female participants were finally qualified for the study and analysis.

The study was conducted under the approval of the Bioethics Committee of the Pomeranian Medical University in Szczecin (Resolution no. KB-012/78/18 of 18.06.2018).

### 4.2. Basic Procedures

All patients underwent a standard interview and physical examination. Anthropometric measurements were taken: height [cm] and weight [kg]. Body mass index (BMI [kg/m^2^]) was calculated from these.

In all participants, bone mineral density was determined in the L1–L4 section of the lumbar spine and in the whole skeleton using dual-energy X-ray absorptiometry (DXA- Dual-energy X-ray absorptiometry). The study was conducted with a GE Lunar Prodigy Advance instrument (Madison, WI, USA) using enCORE software (version 8.8). The results are presented as absolute values (g/cm^2^) and in the form of a Z-score comparing the subject’s score to that of an age-matched control group. A Z-score > −1.0 was considered a normal value, according to the current standards. In the assessment of bone mineralisation, the analysis was performed for BMD values L1–L4 and Z-score L1–L4. According to the Z-score L1–L4 values, the patients were divided into a group with normal bone mineral density (Z-score > −1.0; group A) and a group with reduced BMD (Z-score < −1.0; group B).

Body composition analysis of the study participants was performed using DXA. The instrument used was the GE Lunar Prodigy 14 (Madison, WI, USA) with CoreScan ™ H8801CP automatic software and an automatic whole-body scan using the manufacturer’s original software (Body Composition). The parameters determined were fat mass (FM), lean body mass (LBM) and visceral fat mass (VF). The fat–lean ratio (FLR) was calculated from the results.

Muscle strength testing was carried out according to a strict procedure by measuring hand grip using a hand dynamometer (SAEHAN company—5030J1). In measurements carried out by this method, the value of isometric hand and forearm strength is obtained. The patients remained in a standing position during the examination. The upper limb under test was flexed at the elbow joint at 90 degrees with the arm remaining in contact with the torso. The subjects were asked to squeeze the dynamometer as hard as possible. Three measurements of the grip strength of each hand were taken, and the mean values of the measurements were calculated from the results. The results are expressed in kilograms.

Sclerostin was measured in serum by an enzyme-linked immunosorbent assay (Soluble Sclerostin (Human) ELISA Kit, AVISCERA BIOSCIENCE, INC; catalogue number SK00385–01)). The assay’s properties were as follows: sensitivity (detection limit)  ± 20 pg/mL, a broad detection range (125~4000 pg/mL), an intra-assay precision (Intra-CV) of 4–8% and an inter-assay precision (Inter-CV) of 6–10%. Measurements were performed according to the manufacturer’s instructions.

### 4.3. Statistical Analysis

In the statistical analysis, the quantitative variables obtained were presented as mean, standard deviation (SD), median (M) and upper and lower quartiles. Cross-correlation analysis of values determining bone mineralisation (BMD L1–L4, Z-score, BMC), muscle mass and strength and sclerostin concentration was performed. Due to individual missing data, the number of N valid pairs was reported when calculating the correlation of individual parameters. Due to the diversification of sclerostin concentrations, patients were assigned to four groups, based on the median intermediate result (1163.5 pg/mL: below the test reference range (I =< 125 pg/mL), below the median value (II = 125–1163.5 pg/mL), above the median value (III = 1163.5–16,000 pg/mL) and above the test reference range (IV ≥ 16,000). The normality of the distribution of continuous variables was assessed using the Shapiro–Wilk test. The Brown–Forsythe test was used to check homogeneity of variance. Comparisons between the four groups were performed by one-way ANOVA or Kruskal–Wallis rank ANOVA, depending on the distribution of the variables. The Kruskal–Wallis test was used as a non-parametric post hoc test. Depending on the distribution of variables, Student’s *t*-test or Mann–Whitney U test was used to compare the means of two groups. Quantitative variables were correlated with each other using Pearson’s correlation for variables with a normal distribution and Spearman’s rank correlation whenever the distribution deviated from normal. Correlation coefficient values in the ranges 0.01–0.19, 0.2–0.39, 0.4–0.59, 0.6–0.79, 0.8–0.99 and 1.0 were considered as very weak, weak, moderate, strong, very strong and perfect correlations, respectively. For negative values, the interpretation was similar but of a negative nature. Statistically significant results were considered to be those with a *p*-value < 0.05. Statistical analysis was performed using Statistica 13.3 software (TIBCO Software, Palo Alto, CA, USA). Correlation charts were prepared using the PQStat software (PQStat Software, Poznań, Poland).

## 5. Conclusions

Muscle strength assessment may be an exponent of reduced bone mineral density in young adult women. Sclerostin testing is so far not useful in the clinical assessment of bone mineralisation in young adult women.

## Figures and Tables

**Table 1:** Statistical characteristics of the studied parameters of female study participants.

Variable	*n*	Average	Median	Q1	Q4	SD *
Age [years]	111	23.80	24.00	23.00	25.00	1.793
Body weight [kg]	111	63.48	61.00	56.00	68.00	11.854
Height [cm]	111	168.21	168.00	164.00	173.00	5.967
BMI [kg/m^2^]	111	22.39	21.00	19.50	23.50	4.223
BMD Total	111	1.14	1.13	1.06	1.21	0.096
BMD L_1_–L_4_	111	1.20	1.19	1.11	1.29	0.122
BMD L_1_–L4_z-score_	111	0.22	0.10	−0.50	0.80	1.001
BMC	111	2390.55	2407.00	2220.00	2624.00	351.032
MS—P [kg]	111	27.96	28.30	24.70	31.30	4.395
MS—L [kg]	111	26.38	26.30	23.10	30.40	4.781
FM [g]	111	20,730.95	18,133.00	14,344.00	23,507.00	9305.786
LBM [g]	111	40,966.77	40,770.00	38,004.00	43,630.00	4439.990
FLR	111	0.50	0.45	0.38	0.58	0.180
VF [g]	111	273.44	161.00	60.00	309.00	355.636
Sclerostin [pg/mL]	111	2136.37	125.00	125.00	263.00	4989.928

MS—P—right hand muscle strength, MS—L—left hand muscle strength, SD—standard deviation, MC—body mass, FM—fat mass, LBM—lean soft tissue mass, FLR—fat–lean ratio, VF—visceral fat mass, BMD Total—total bone mineral density, BMD L1–L4—bone mineral density of the L1–L4 segment, BMD L1–L4_z-score_—bone mineral density Z-score (standard deviation from mean value), BMC—bone mineral content, * *n*—number of variables, Q1—lower quartile, Q4—upper quartile, SD—standard deviation.

**Table 2:** Statistical characteristics and comparison of study parameters of female participants in groups A (with normal bone mineral density) and B (with reduced bone mineral density).

Variable	Group A			Group B			Value *p*
	*n*	Mean (SD/Median)	Q1–Q4	*n*	Mean (SD/Median)	Q1–Q4	
MS—P	95	28.23 (4.52/28.40)	24.80–31.50	16	26.36 (3.21/26.15)	24.10–32.10	>0.1
MS—L	95	26.73 (4.38/26.70)	23.20–30.50	16	24.27 (6.49/24.19)	21.50–28.30	>0.1
FM	95	20,198.45 (8763.76/18,166.00)	14,344.00–22,927.00	16	23,892.69 (11,886.51/17,930.00)	15,531.00–32,191.50	>0.1
LBM	95	41,032.59 (4404.43/41,287.00)	38,035.00–43,612.00	16	40,576.00 (4775.81/39,421.50)	36,094.00–45,777.50	>0.1
Sclerostin [pg/mL]	95	1950.59 (4740.95/125.0)	125.00–244.00	16	3239.45 (6342.35/125.00)	125.00–1228.12	>0.1

MS—R—muscle strength of right hand, MS—L—muscle strength of left hand, FM—fat mass, LBM—lean body mass, *n*—number of variables, SD—standard deviation, Q1—lower quartile, Q4—upper quartile.

**Table 3:** Relationship between BMD L1–L4 bone mineral density and the parameters studied.

Variable	*n*	R	*p*
MS—P [kg]	111	0.3090	0.0009
MS—L [kg]	111	0.3683	0.0000
FM [g]	111	0.2788	0.0030
LBM [g]	111	0.9214	0.0000
FLR	111	0.2042	0.0315
VF [g]	111	0.1586	0.0963
Sclerostin [pg/mL]	111	0.0204	0.8314

MS—P—muscle strength of the right hand, MS—L—muscle strength of the left hand, FM—fat mass, LBM—lean soft tissue mass, FLR—fat–lean ratio, VF—visceral fat mass, *n*—number of variables, R—Spearman’s/Pearson’s rank correlation coefficients, *p*—*p*-value.

**Table 4:** Correlation of left and right upper limb muscle strength with selected parameters.

	Muscle Strength Left Upper Limb			Muscle Strength Right Upper Limb		
Variable	*n*	R	*p*	*n*	R	*p*
BMD Total	111	0.3246	0.0005	111	0.1692	0.0759
BMD L1–L4	111	0.3683	0.0001	111	0.2872	0.0020
BMD L1–L4_z-score_	111	0.3086	0.0010	111	0.2520	0.0080
BMC	111	0.3864	0.0000	111	0.3023	0.0013
FM [g]	111	0.1379	0.1491	111	0.0126	0.8958
LBM [g]	111	0.3618	0.0001	111	0.2434	0.0110
FLR	111	0.0407	0.6713	111	−0.0787	0.4118
VF [g]	111	0.0206	0.8301	111	−0.0815	0.3951
Sclerostin [pg/mL]	111	0.1452	0.1285	111	0.1561	0.1018

FM—fat mass, LBM—lean soft tissue mass, FLR—fat–lean ratio, VF—visceral fat mass, BMD Total—total bone mineral density, BMD L1–L4—bone mineral density of the L1–L4 segment, BMD L1–L4_z-score_—bone mineral density Z-score (standard deviation from mean value), BMC—bone mineral content, *n*—number of variables, R—Spearman’s/Pearson’s rank correlation coefficients, *p*—*p*-value.

**Table 5:** Correlation of lean tissue mass (LBM) with study parameters.

Variable	*n*	R	*p*
BMD Total	111	0.615	0.000
BMD L1–L4	111	0.301	0.001
BMD L1–L4_z-score_	111	−0.023	0.814
BMC	111	0.771	0.000
FM [g]	111	0.526	0.000
FLR	111	0.286	0.002
VF [g]	111	0.303	0.001
Sclerostin [pg/mL]	111	0.186	0.051

FM—fat mass, LBM—lean body mass, FLR- fat–lean ratio, VF—visceral fat mass, BMD Total—total bone mineral density, BMD L1–L4—bone mineral density of the L1–L4 segment, BMD L1–L4_z-score_—bone mineral density Z-score (standard deviation from mean value), BMC—bone mineral content, *n*—number of variables, R—Spearman’s/Pearson’s rank correlation coefficients, *p*—*p*-value.

## Data Availability

Data are available on special request after contacting author (M.P.-W.).
